# Ancient DNA Resolves Identity and Phylogeny of New Zealand's Extinct and Living Quail (*Coturnix sp*.)

**DOI:** 10.1371/journal.pone.0006400

**Published:** 2009-07-28

**Authors:** Mark Seabrook-Davison, Leon Huynen, David M. Lambert, Dianne H. Brunton

**Affiliations:** 1 Ecology and Conservation Group, Institute of Natural Sciences, Massey University, Auckland, New Zealand; 2 Institute of Natural Sciences, Massey University, Auckland, New Zealand; 3 Griffith School of Environment and School of Biomolecular and Physical Sciences, Griffith University, Nathan, Australia; McGill University, Canada

## Abstract

**Background:**

The New Zealand quail, *Coturnix novaezealandiae*, was widespread throughout New Zealand until its rapid extinction in the 1870's. To date, confusion continues to exist concerning the identity of *C. novaezealandiae* and its phylogenetic relationship to *Coturnix* species in neighbouring Australia, two of which, *C. ypsilophora* and *C. pectoralis*, were introduced into New Zealand as game birds. The Australian brown quail, *C. ypsilophora*, was the only species thought to establish with current populations distributed mainly in the northern part of the North Island of New Zealand. Owing to the similarities between *C. ypsilophora*, *C. pectoralis*, and *C. novaezealandiae*, uncertainty has arisen over whether the New Zealand quail is indeed extinct, with suggestions that remnant populations of *C. novaezealandiae* may have survived on offshore islands.

**Methodology/Principal Findings:**

Using fresh and historical samples of *Coturnix sp*. from New Zealand and Australia, DNA analysis of selected mitochondrial regions was carried out to determine phylogenetic relationships and species status. Results show that *Coturnix sp*. specimens from the New Zealand mainland and offshore island Tiritiri Matangi are not the New Zealand quail but are genetically identical to *C. ypsilophora* from Australia and can be classified as the same species. Furthermore, *cytochrome b* and *COI* barcoding analysis of the New Zealand quail and Australia's *C. pectoralis*, often confused in museum collections, show that they are indeed separate species that diverged approximately 5 million years ago (mya). Gross morphological analysis of these birds suggests a parallel loss of sustained flight with very little change in other phenotypic characters such as plumage or skeletal structure.

**Conclusion/Significance:**

Ancient DNA has proved invaluable for the detailed analysis and identification of extinct and morphologically cryptic taxa such as that of quail and can provide insights into the timing of evolutionary changes that influence morphology.

## Introduction

Quail in the *Coturnix* complex are widely distributed throughout Australasia, Asia, and various Pacific Islands [12]. All species are well established in their home ranges except for the New Zealand quail *C. novaezealandiae* [3], [4] which, although common until the mid-1800s, declined rapidly to extinction by the late-19^th^ century [5]–[7].

We wished to investigate the phylogenetic relationship between the two extant Australian quail species, *C. ypsilophora* and *C. pectoralis*, and the New Zealand quail *C. novaezealandiae*, as well as an extant New Zealand quail (*Coturnix sp*.) that has been resident on Tiritiri Matangi Island for over 100 years [8]. Both the Australian brown quail, *C. ypsilophora*, and stubble quail, *C. pectoralis*, are widespread throughout Australia but are particularly abundant in the continent's southern and eastern regions. Early records [3], [4] from New Zealand suggest that *C. novaezealandiae* was widespread throughout the archipelago but declined rapidly in the mid-1800s as a result of large-scale habitat burning and predation by dogs, cats, and rats and was declared extinct by 1875 [2], [9].

**Figure 1:**
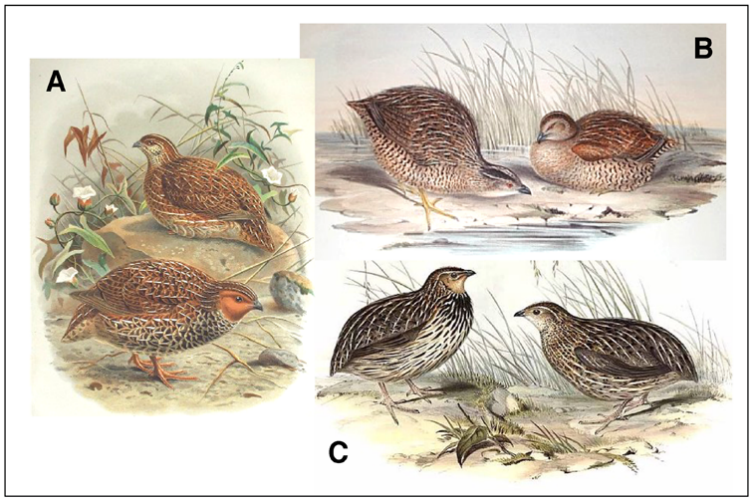
Illustrations of Australasian quail. Each is a breeding pair with males on the left. A. *C. novaezealandiae* (image from; A History of the Birds of New Zealand, Sir Walter Buller, 1888) B. *C. ypsilophora*. C. *C. pectoralis* (images from; Birds of Australia, Volume V, John Gould, 1848).

Phenotypic similarity between the three quail species has led to confusion in historical reports and museum records ([2]; Figure 1). Distinguishing *C. pectoralis* from *C. novaezealandiae* is particularly difficult by morphology alone and has led to the mislabelling of numerous museum skins (B. Gill, Auckland Museum, pers comm. 2006). Taxonomic confusion was compounded by the release of *C. ypsilophora* and *C. pectoralis* into New Zealand during the years 1866–1872 [10], [11] and the subsequent reported decline of remnant populations of *C. novaezealandiae*, possibly aided by the spread of diseases from the introduced game birds [13]. The isolation and relatively untouched habitats on New Zealand's off-shore islands such as Tiritiri Matangi has led to speculation that some populations of *C. novaezealandiae* may have survived to present day.

Using DNA sequences from selected mitochondrial regions we determine the identity of New Zealand quail species, and clarify its relationship with other Australasian quail as well as Asian and Pacific *Coturnix* species and other genera in the *Phasianidae* family.

## Results

**Figure 2:**
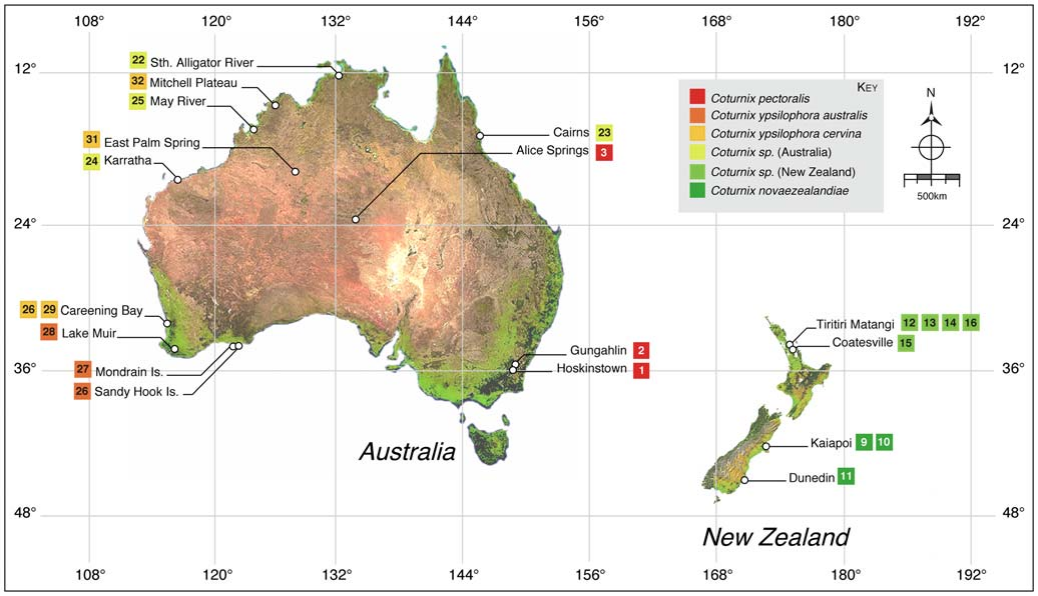
*Coturnix sp*. samples used for which location data were available. Nearest location to samples used are shown. Numbers correspond to those shown in Table 1. Each square represents a single sample. Colours delineate species and sub-species (see Key).

**Figure 3:**
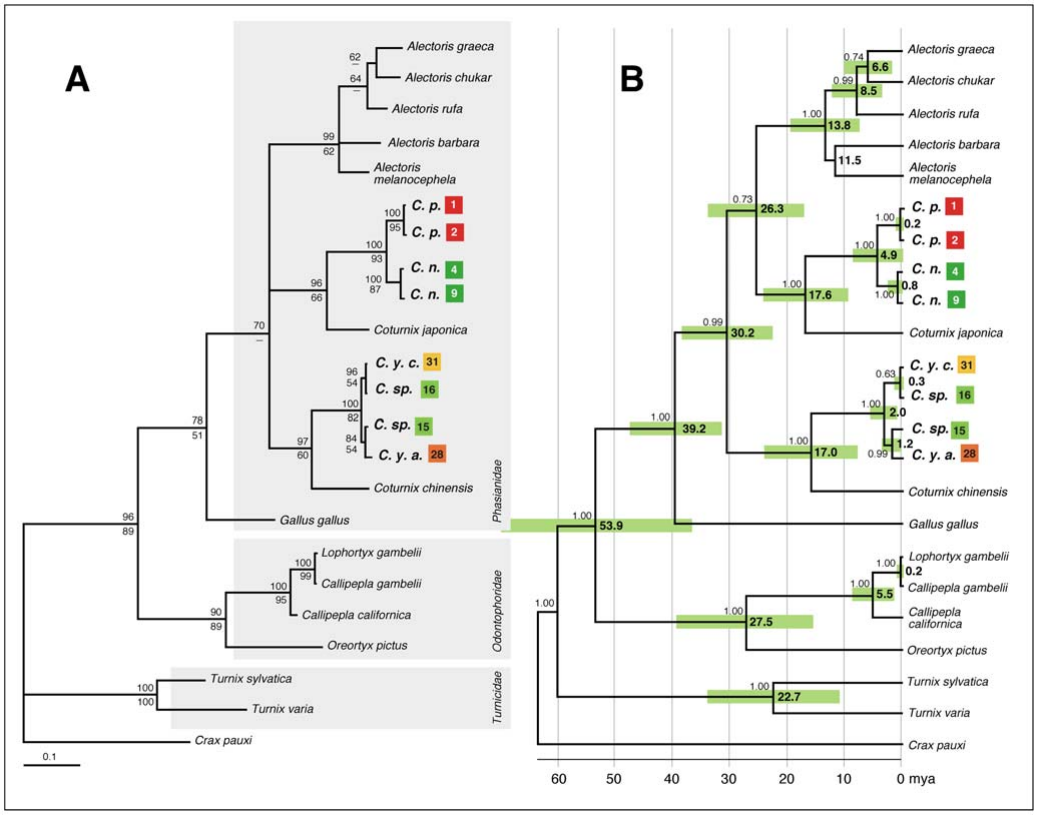
Phylogenetic relationships and split times between *Coturnix sp*. A. A distance neighbour-joining tree was constructed with 456 bp of mitochondrial *cytochrome b* sequence in PAUP* v4.0beta10 using the TrN+I+G model of nucleotide substitution where the proportion of invariable (I) sites was calculated to be 0.438 and the Gamma distribution shape parameter (G) was 0.755. Node bootstrap values were determined using both Distance (above line) and ML (below line) -based methods. Only values greater than 50% are shown. *Crax Pauxi* from the *Cracidae* family was used as outgroup. B. BEAST v1.4.8 maximum clade credibility tree. Divergence times are given in millions of years ago (mya; 95% HPD indicated by the green boxes). Node posterior probability values were calculated in BEAST v1.4.8 and values greater than 0.6 are shown above the branch lines. Abbreviations, colours, and numbers are as outlined in Figure 2.

Despite detailed analysis using the mitochondrial genes *cytochrome b* (*cytb*) and *NADH dehydrogenase* 2 (*ND2*), the general phylogeny of the *Phasianidae* family is largely unresolved [14]–[17]. However, using either of these genes has been useful for grouping closely related quail species in their respective genus [15]. We show, using a number of samples (Figure 2), and 456 base pairs (bp) of the mitochondrial *cytb* gene, that the New Zealand quail *C. novaezealandiae*, is a likely sister species of *C. pectoralis*, endemic to Australia, with both being very closely related to the Japanese quail *C. japonica* (Figure 3A). Furthermore, little difference could be detected between *cytb* sequences from the Australian quail *C. ypsilophora*, and extant *Coturnix sp*. present in New Zealand, suggesting that these are likely to be the same species, and are a sister species to the king quail *C. chinensis*. *Cytb* sequence from the New Zealand based Californian quail was, as expected, identical to that from *Callipepla californica* (data not shown).

Using the DNA barcoding region (approximately 600 bp of the 5′ terminus of the mitochondrial *cytochrome oxidase I* gene, *COI*; [18]) we tested the species status of *C. novaezealandiae*, *C. pectoralis*, *C. ypsilophora*, and extant New Zealand quail. *COI* has, to date, successfully distinguished over 3000 avian taxa, including *C. japonica* and *C. chinensis*, by simply measuring *COI* sequence divergence within or between species (http://www.boldsystems.org/views/taxbrowser.php?taxid=51). Individuals differing by less than 2% over this DNA region have been shown to belong to the same species (http://www.boldsystems.org/views/taxbrowser.php?taxid=51). *COI* divergence between *C. pectoralis* and *C. novaezealandiae* was calculated to be 3.0%. This high value, in addition to the time these species have spent in geographic isolation suggests that they are very likely to be separate taxa. In contrast, *COI* sequences for *C. ypsilophora* and extant New Zealand quail differed by less than 0.78%, suggesting that they belong to the same taxon.

**Figure 4:**
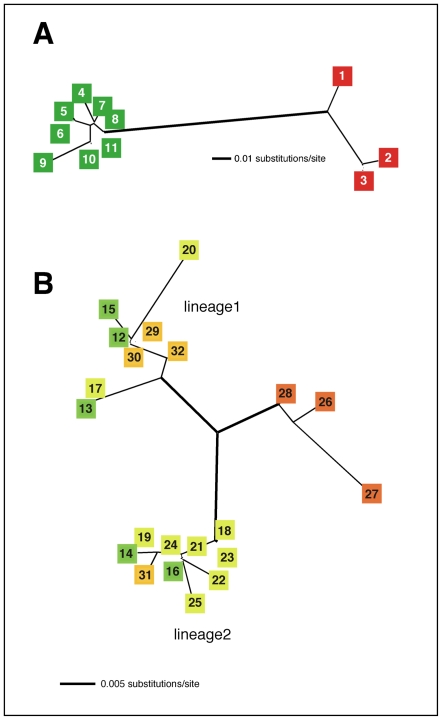
Network analysis of Australasian *Coturnix sp*. An unrooted distance neighbour-joining tree was constructed with 213 bp of mitochondrial HVR1 sequence in PAUP*4.0beta10 using both the HKY85+G (G = 0.150); Ti/tv = 3.0(A) or K81+I (I = 0.79) (B) model of substitution [24]. A. *C. novaezealandiae* and *C. pectoralis*. B. *C. ypsilophora* and extant New Zealand quail. Colours and numbers correspond to sample descriptions in Figure 2.

Detailed analysis of New Zealand and Australian quail populations was carried out using mitochondrial sequence from a region, HVRI, of the highly variable d-loop and show that the extant New Zealand quail population is genetically identical to that of the Australian brown quail, in particular to the subspecies *C. ypsilophora cervina*. An unrooted phylogram constructed from HVRI sequences suggests the existence of two lineages within this subspecies, each distinct from the subspecies *C. ypsilophora australis*, and shows no clear geographic structure (Figure 2 and Figure 4).

Divergence times for New Zealand and Australian quail species were determined in BEAST v1.4.8 [19] calibrated with a *Gallus gallus*/*Coturnix chinensis* split time, using *cytb* sequences, of 40.4±4.1 mya (Figure 3B) [20]. The divergence time between *C. novaezealandiae* and *C. pectoralis* was estimated to be 4.9 mya (95% highest posterior density interval (HPD); 2.2–7.9 mya). Similarly a split time for lineage 1 and lineage 2 of *C. ypsilophora cervina* was shown to be 2.0 mya (95%HPD; 0.7–3.6 mya) and the divergence time of *C. ypsilophora cervina* lineage 1 and *C. ypsilophora australis* was calculated to be 1.2 mya (95%HPD 0.2–2.3 mya).

## Discussion

The results presented clarify queries concerning the phylogenetic relationship between Australian and New Zealand *Coturnix* species. Confirmation has been provided from both mitochondrial HVR1 and *cytb* sequences that the New Zealand quail *C. novaezealandiae* is closely related to the Australian stubble quail *C. pectoralis* and does not show a close relationship with the Australian brown quail *C. ypsilophora* (data for the latter not shown using HVR1 sequences). Furthermore, New Zealand's extant quail from the offshore Island Tiritiri Matangi is *C. ypsilophora* and not *C. novaezealandiae*. Questions remain whether *C. novaezealandiae* has survived on other offshore islands such as the Three Kings Islands, approximately 55 km northwest of New Zealand's most northern tip, Cape Reinga. However, close examination of Official Acclimatisation Society and museum records [10], [11] suggests that this is unlikely. All quail specimens from distant offshore islands recorded by the Auckland Museum, Acclimatisation Society or New Zealand Ornithological Society are listed as *C. ypsilophora* [32]. Divergence time estimates indicate that the common ancestor of *C. pectoralis* and *C. novaezealandiae* existed approximately 5 mya and in the absence of an Australian/New Zealand land-bridge since the Late Cretaceous (∼65–145 mya) [12], was likely to be capable of sustained long-range flight between the two land masses. Both *C. pectoralis* and *C. novaezealandiae* appear to have lost this trait independently in preference to clear changes in other phenotypic traits such as plumage or skeletal structure.

The Australian brown quail is distributed throughout Australia, New Guinea, and the Lesser Sunda Islands. Morphological examination of museum specimens by Marchant and Higgins (1993) [2], suggests the existence of as many as seven polytypic variants and three subspecies of *C. ypsilophora*. However, Marchant and Higgins (1993) [2] have called for a review of the taxonomy of *C. ypsilophora* but in the absence of this analysis they nominate *C. ypsilophora ypsilophora* for the Tasmanian subspecies, possibly *C. ypsilophora cervina* for north Australia, and *C. ypsilophora australis* for the rest of Australia. The unrooted phylogenetic tree of HVRI sequences presented here, shows a clear split between *C. ypsilophora australis* and *C. ypsilophora cervina* (Figure 4B), and suggests that two lineages exist for the latter subspecies. Both *C. ypsilophora cervina* lineages are present in New Zealand and these are most likely derived from the game birds that were introduced in the mid-19^th^ century [10].

Results from a phylogenetic analysis using *cytb* sequences show a close relationship of *C. novaezealandiae* and *C. pectoralis* with the Japanese quail, *C. japonica* (Figure 3A). The Japanese quail is resident in the Japanese archipelago but has a recorded distribution throughout China and Korea. Unlike Australasian quail, it is a migratory species that breeds in Manchuria, southeast Siberia, and northern Japan with a feeding, over-wintering stage in China, Korea, and southern Japan [21], [23]. Interestingly, *C. japonica* has never been found further south through the Cambodian, Malaysian Peninsula or through the Indonesian archipelago to Australia and New Guinea. Phylogenetic analysis also shows a close relationship between *C. ypsilophora* and *C. chinensis*, suggesting that they are sister species. The current distribution of these two species overlaps throughout Australia, Papua New Guinea, and Indonesia. From all *Coturnix* species in Australasia, Asia, the Pacific Islands, and India, the only insular taxon is (now extinct) *C. novaezealandiae*.

All Australasian *Coturnix sp*. are almost indistinguishable by plumage alone, with all sporting brown feathers with vertical buff streaking. Although nearly all group together genetically, the Australian brown quail *C. ypsilophora* is unusual in that it is most closely related to the highly colourful *C. chinensis*.

Molecular analysis has proven to be a valuable tool in understanding phylogenetic relationships between species. The ability to extract DNA from ancient tissue is now becoming routine, making it possible to assign species names to unknown museum specimens as well as constructing phylogenies for species complexes. The resultant information is not only useful for taxonomic purposes [22] and evolutionary analysis, but can have important implications for conservation [29], [30]. Taxonomically, specimens held in museum collections can be correctly labelled. In conservation terms, phylogenetic lineages can be better understood when species assemblages are being considered for restoration.

## Materials and Methods

### Sample collection

Samples used in this work are outlined in Table 1. All samples were stored at a dedicated ancient DNA laboratory at the Institute of Natural Sciences (INS) Massey University, Auckland, New Zealand. Fresh tissue was kept at −80°C, whilst feathers, bone, and footpads were kept at room temperature.

**Table 1:** Quail samples.

Sample ID	Fig ID	Tissue	Species	Location (map co-ordinates)	Collector, date, notes
NTM_T.1207	1	fp	*C. p.*	Hoskinstown, NSW, Aust.	B. Brown, 12/05/1976
NTM_T.1209	2	fp	*C. p.*	Gungahlin, ACT, Aust.	W. H. Ewers, 15/04/1963
NTM_T.2453	3	fp	*C. p.*	Alice Springs, NT (23°42'00''S 133°53'00''E), Aust.	C. H. Brown, 05/03/1955
OM_Av8209	4	bn	*C. n.*	Otago, NZ?	
OM_Av8114	5	bn	*C. n.*	Otago, NZ?	
CM_Av2349	6	fp	*C. n.*	Canterbury, NZ?	z204 0.1121.0 immature
CM_Av2350	7	fp	*C. n.*	Canterbury, NZ?	
CM_Av2351	8	fp	*C. n.*	Canterbury, NZ?	chick
CM_Av1665	9	fp	*C. n.*	Kaiapoi, Canterbury, NZ.	Buller Collection, 1859
CM_Av1666	10	fp	*C. n.*	Kaiapoi, Canterbury, NZ.	Buller Collection, 1859
CM_Av33172	11	fp	*C. n.*	Dunedin, NZ?	ex Norman Potts
TtM_A1	12	bl	*C. sp.*	Wharf rd., Tiritiri Matangi, Ak, NZ	M. Seabrook-Davison, 04/12/2006
TtM_A3	13	ft	*C. sp.*	Fishermans Bay, Tiritiri Matangi, Ak, NZ	M. Seabrook-Davison, 05/12/2006
TtM_A4	14	ft	*C. sp.*	Fishermans Bay, Tiritiri Matangi, Ak, NZ	M. Seabrook-Davison, 05/12/2006
Cv_17.10	15	ft	*C. sp.*	Coatesville, Ak, NZ	M. Seabrook-Davison, 17/10/2005
TtM_7.7	16	ft	*C. sp.*	Tiritiri Matangi, Ak, NZ	M. Seabrook-Davison,
MAGNT_T970	17	fp	*C. y.*	Northern Territory, Aust.?	
MAGNT_T1027	18	fp	*C. y.*	Northern Territory, Aust.?	
MAGNT_T1588	19	fp	*C. y.*	Northern Territory, Aust.?	
MAGNT_T2454	20	fp	*C. y.*	Northern Territory, Aust.?	
MAGNT_T4102	21	fp	*C. y.*	Northern Territory, Aust.?	
MWA_A28208	22	fp	*C. y.*	Sth Alligator River, NT (13°05'00''S 132°18'00''E), Aust.	18/10/1902
MWA_A28213	23	fp	*C. y.*	Cairns, QL (16°55'00''S 145°46'00''E), Aust.	00/11/1889
MWA_A34652	24	fp	*C. y.*	Karratha, WA (20°44'00''S 116°52'00''E), Aust.	28/08/2000
MWA_A34751	25	fp	*C. y*	May River, WA (17°17'00''S 123°59'00''E), Aust.	03/10/1996
MWA_A15336	26	fp	*C. y. a.*	Sandy Hook Island, WA (34°02'00''S 122°00'00''E), Aust.	19/11/1904
MWA_A15338	27	fp	*C. y. a.*	Mondrain Island, WA (34°08'00''S 125°15'00''E), Aust.	28/04/1906
MWA_A20107	28	fp	*C. y. a.*	Lake Muir, WA (34°29'00''S 116°40'00''E), Aust.	01/04/1986
MWA_A12637	29	fp	*C. y. c.*	Careening Bay, WA (32°13'58''S 115°40'58''E), Aust.	26/06/1973
MWA_A12638	30	fp	*C. y. c.*	Careening Bay, WA (32°13'58''S 115°40'58"E), Aust.	26/06/1973
MWA_A13879	31	fp	*C. y. c.*	East Palm Spring, WA (19°20'00"S 128°20'00"E), Aust.	24/06/1975
MWA_A15741	32	fp	*C. y. c.*	Mitchell Plateau, WA (14°48'00"S 125°50'00"E), Aust.	27/09/1978

Fig ID refers to the sample identification number used in subsequent figures. Abbreviations are: MWA – Museum of Western Australia, MAGNT – Museum & Art Gallery of the Northern Territory, OM – Otago Museum, CM – Canterbury Museum, fp – footpad, bn – bone, ft – feather, bl – blood, *sp. - species*, *C. p. - Coturnix pectoralis, C. n. - Coturnix novaezealandiae, C. sp – Coturnix species, C. y. – Coturnix ypsilophora, C. y. c. – Coturnix ypsilophora cervina, C. y. a. – Coturnix ypsilophora australis*. States in Australia (Aust.) are represented as; NSW – New South Wales, QL – Queensland, ACT – Australian Capital Territory, WA – Western Australia, NT – Northern Territory. New Zealand (NZ) locations are; Ak – Auckland, TtM – Tiritiri Matangi, Cv – Coatesville, ? – likely location.

Blood was taken from live quail immediately after they were caught. Less than 1% of the total blood volume of quail was taken from the wing brachial artery and stored in Queens lysis buffer [24] at 4°C. Feathers were collected from both fresh and preserved specimens. To ensure that adequate genetic material was obtained, the whole feather including the shaft and tissue contained in the hollow end of the shaft was removed from the bird. Feathers were collected in paper envelopes and stored in a dry humidity free facility. Footpad samples were taken from frozen specimens when required. Preserved footpad samples were obtained from museum specimens throughout New Zealand and Australia.

### DNA extraction

DNA was extracted using proteinase K digestion and column purification [25], [26]. Briefly, tissue samples, footpads, ∼2 ul of blood, or feather bulbs were incubated in 200 ul of SET buffer (50 mM Tris-Cl pH 8.0, 5 mM EDTA, 50 mM NaCl) supplemented with dithiothreitol (DTT) to 50 mM, sodium dodecyl sulphate (SDS) to 1% (w/v) and approximately 50 ug of proteinase K.

Samples were incubated with rotation overnight at 56°C, and the DNA was extracted with an equal volume of phenol:chloroform (1∶1), before being purified using a QIAQuick® DNA purification kit (Qiagen). Purified DNA was eluted in 100 ul of elution buffer and stored at –20°C. DNA was extracted from bone in the same way, except for an initial decalcification of the bone shavings (∼10–50 mg) by incubation at room temperature, with rotation, overnight in ∼500 ul of 0.5 M EDTA pH 8.0.

### DNA amplification and sequencing

A number of quail mitochondrial genes were investigated to provide information on phylogeny (*cytochrome b*), species status (*cytochrome oxidase subunit I*), and genetic relationships within quail species (HVRI). These regions were selected because each are characterized by appropriate rates of molecular change in relation to the problem under investigation. All Amplified DNA products were obtained using the same conditions. Approximately 1 ul (∼1 – 20 ng) of DNA was amplified by polymerase chain reaction (PCR) in 10 ul volumes containing 50 mM Tris-Cl pH 8.8, 20 mM (NH_4_)_2_SO_4_, 2.5 mM MgCl_2_, 1 mg/ml BSA, 200 uM of each dNTP, 40 ng of each primer, and ∼0.3 U of platinum Taq (Invitrogen). The reaction mix was overlaid with mineral oil and subjected to amplification in a Hybaid OmniGene thermal cycler using the following parameters: 94°C for 2 min (×1), 94°C for 20 sec, 54°C for 20 sec, 72°C for 20 sec (×15), and then 94°C for 20 sec, 50°C for 20 sec, and 72°C for 20 sec (×35). Amplified DNAs were detected by agarose gel electrophoresis in Tris-borate-EDTA buffer (TBE), stained with 50 ng/ml ethidium bromide in TBE, and then visualized over UV light. Positive amplifications were purified by centrifugation through ∼40 ul of dry Sephacryl™ S300HR and then sequenced at the Allan Wilson Centre Genome Sequencing Service using Applied Biosystems (ABI) BigDye® Terminator v3.1 chemistry and an ABI3730 Genetic Analyzer. Amplification and sequencing primers are shown in Table 2.

**Table 2:** Oligonucleotide primers.

Primer name	Sequence (5′–3′)	Mitochondrial target [5′ binding site]	Tm (°C)
ctcF1n	TCGTGCATACATTTATATTCCACA	Hypervariable region I (HVRI) [76]	63
ctcR1n	TGATACGACGAGCATAACCAA	Hypervariable region I (HVRI) [333]	63
cCOIF1ii	AAGGACTACAGCCTAAC	*cytochrome C oxidase* I (*COI*) [6506]	48
cCOIR1ii	ACGAGTCAATTTCCGAAG	*cytochrome C oxidase* I (*COI*) [6798]	58
cCOIF2ii	GTAATyGTCACAGCCCATG	*cytochrome C oxidase* I (*COI*) [6716]	59
cCOIR2ii	GAAAAGATGGCTAGrTCTAC	*cytochrome C oxidase* I (*COI*) [6996]	52
cCOIF3iii	TTAGCyGGyAACCTAGCCCA	*cytochrome C oxidase* I (*COI*) [6944]	63
cCOIR3iii	AGGGTCGAAGAATGTGGTGTT	*cytochrome C oxidase* I (*COI*) [7216]	61
ccytBF1ii	GAAATGTACAGTACGGCTGACT	*cytochrome b* (*cytb*) [15018]	60
ccytBR1ii	CTGAGAATAGGTTTGTGATGAC	*cytochrome b* (*cytb*) [15260]	58
ccytBF2	CCATTCCTAATCGCAGGAA	*cytochrome b* (*cytb*) [15362]	63
ccytBR2	ATTGAACGTAGGATGGCGTA	*cytochrome b* (*cytb*) [15657]	62

All primers were ordered dry from Sigma-Genosys, resuspended in milliQ water to 2 ug/ul and stored at −20°C. Primer binding site positions (5′ terminus) were determined using the complete mitochondrial sequence of *Coturnix chinensis*, GenBank accession number: AB073301.

### Ancient DNA

Both “fresh” and historical samples were treated using criteria set for the retrieval of DNA from ancient tissues. All DNA extractions were carried out in a physically separate, dedicated ancient DNA laboratory at Massey University away from the main laboratory where amplifications were performed. Sequences were obtained in both directions from separate amplifications and in most cases from multiple extractions. Sequences from several samples were verified by extraction and amplification at the Griffith University Ancient DNA Facility, Nathan, Australia.

### Phylogenetic analysis

DNA sequences were edited and aligned in Sequencher™. All sequences have been deposited in GenBank with accession numbers: mitochondrial HVRI, GQ150346-GQ150377; *cytochrome b* (*cytb*), GQ150388-GQ150395; *cytochrome oxidase subunit I* (*COI*), GQ150378-GQ150387. The most likely evolutionary model for each set of sequences was determined using the Akaike Information Criterion (AIC) in ModelTest v3.7 [27]. Optimal phylogenetic trees were constructed using the distance neighbour-joining method in PAUP* v4.0beta10 [28]. Node bootstrap values for the tree constructed using *cytb* sequences were determined in PAUP* v4.0beta10 using both Maximum Likelihood (ML; 1000 “fast” stepwise-addition replicates) and Distance (1000 full heuristic replicates) algorithms. Node ages were determined for the same tree using BEAST v1.4.8 [19] and the GTR + G model of nucleotide substitution. A relaxed uncorrelated lognormal clock was stipulated and Yule process was set as tree prior. A normal prior distribution of 40.4±4.1 mya for the *Gallus gallus*/*Coturnix chinensis* split was used to calibrate the tree [20]. Three independent runs of 10 million trees each were carried out with one tree sampled every 200 generations and the first 12,500 trees discarded as “burn-in”. Chain convergence was analysed for each run using Tracer v1.4 [31] and the most probable maximum clade credibility tree was found using TreeAnnotator v1.4.8. The resultant tree was visualized and edited in FigTree v1.2.2. *Cytochrome b* sequences retrieved from GenBank for tree construction were: *Coturnix japonica*, AF119094; *Coturnix chinensis*, AB073301; *Alectoris graeca*, Z48772; *Alectoris chukar*, AM850828; *Alectoris rufa*, AM850844; *Alectoris melanocephela*, Z48773; *Alectoris barabara*, Z48771; *Oreortyx pictus*, AF252860; *Gallus gallus*, AP003322; *Lophortyx gambelii*, L08382; *Callipepla gambelii*, DQ485889; *Callipepla californica*, AB120131; *Turnix sylvatica*, DQ385232; *Turnix varia*, AF168104; and *Crax pauxi*, AF068190.
